# Identification of QTLs controlling gene expression networks defined *a priori*

**DOI:** 10.1186/1471-2105-7-308

**Published:** 2006-06-16

**Authors:** Daniel J Kliebenstein, Marilyn AL West, Hans van Leeuwen, Olivier Loudet, RW Doerge, Dina A St Clair

**Affiliations:** 1 University of California-Davis, Department of Plant Sciences, Mail Stop 3, One Shields Ave, Davis, CA 95616-8780, USA; 2 INRA, Station de Génétique et d'Amélioration des Plantes, Centre de Versailles, 78026Versailles, France; 3 Purdue University, Department of Statistics, Mathematical Sciences Building, 150 North University Street, West Lafayette, IN 47907-2067, USA

## Abstract

**Background:**

Gene expression microarrays allow the quantification of transcript accumulation for many or all genes in a genome. This technology has been utilized for a range of investigations, from assessments of gene regulation in response to genetic or environmental fluctuation to global expression QTL (eQTL) analyses of natural variation. Current analysis techniques facilitate the statistical querying of individual genes to evaluate the significance of a change in response, also known as differential expression. Since genes are also known to respond as groups due to their membership in networks, effective approaches are needed to investigate transcriptome variation as related to gene network responses.

**Results:**

We describe a statistical approach that is capable of assessing higher-order *a priori *defined gene network response, as measured by microarrays. This analysis detected significant network variation between two *Arabidopsis thaliana *accessions, Bay-0 and Shahdara. By extending this approach, we were able to identify eQTLs controlling network responses for 18 out of 20 *a priori*-defined gene networks in a recombinant inbred line population derived from accessions Bay-0 and Shahdara.

**Conclusion:**

This approach has the potential to be expanded to facilitate direct tests of the relationship between phenotypic trait and transcript genetic architecture. The use of *a priori *definitions for network eQTL identification has enormous potential for providing direction toward future eQTL analyses.

## Background

Many phenotypic traits, ranging from disease susceptibility to development, are quantitative in nature and are studied in both animals and plants via quantitative trait locus (QTL) mapping [1-3]. QTLs are regions of the genome associated with phenotypic variation for a trait. These regions may or may not contain genes that, when differentially expressed, control the associated phenotypic variation. One approach that explores the relationship of phenotypic trait variation with transcriptome variation employs microarrays to survey global gene expression across a sample of individuals from a segregating population, and then maps expression QTLs (eQTLs) [4-7]. An inventory of eQTLs representing a population or species may provide the necessary information required for identifying genes that control quantitative phenotypes. Categorizing eQTLs has the potential to enable reverse (natural variation) genetics approaches for the identification of genes controlling quantitative traits, and may also help to enhance the rate of QTL cloning [8].

Global eQTL analyses also allow evolutionary biologists and geneticists a broader view of molecular complexities. For example, what is the level of *cis *versus *trans *polymorphism controlling gene expression in a species, and which is more likely to cause a phenotypic alteration? Initial observations from global transcriptome QTL mapping studies indicate that eQTLs are located in *cis *or *trans *relative to the gene's physical position, but neither the *cis *nor *trans *eQTL positions have been directly linked to phenotypic consequences [4,9,10]. Furthermore, at what regulatory level in the global gene expression networks are the *trans *polymorphisms typically acting? Are they upstream in a regulatory network, and hence control large numbers of genes in *trans*? Or, are they downstream in a network and thereby affect only a limited number of genes? Finally, how is transcript variation and heritability related to the resulting phenotypic variation and heritability [11]? Addressing these questions requires the classification of eQTLs with respect to their *cis *and *trans *effects, a quantification of the number of genes that *trans *eQTLs control, and an assessment of whether the genes controlled by a single *trans *eQTL are functionally related.

**Figure 1:**
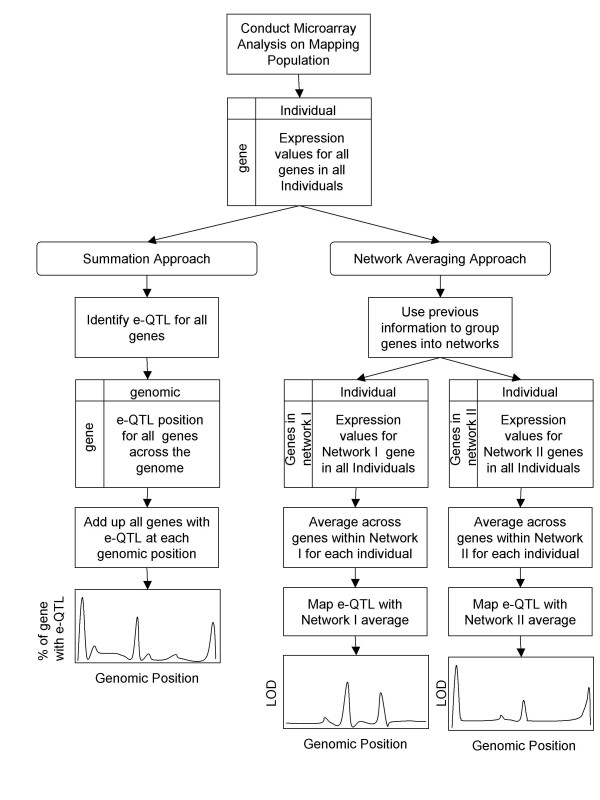
**Network analysis of microarray data**. A flow-chart describing the summation approach and the network averaging approach. The summation approach is previously described in the literature (see references in the text). The network averaging approach is the method developed and utilized in this study.

One goal of global eQTL analysis is to identify loci controlling the expression variation of gene networks associated with various biological functions. One approach [4,6] is to generate a mapping population, assess global gene expression using microarrays, and identify eQTLs controlling the expression of each gene via individual statistical analyses. The eQTL locations from these individual analyses for all genes are then superimposed to identify common regions that control the expression of a large number of genes, i.e. contain 'broad effect' eQTLs. This method is hereafter referred to as the *summation approach *(Figure 1 – summation approach) [4,12]. It requires that genes exhibit expression variation and that there is both sufficient biological and technical replication, but it does not require the assignment of *a priori *network information. Specifically, current approaches require *a posteriori *tests to assess whether the genes controlled by an identified *trans *eQTL regions share a common biological function (e.g., a metabolic pathway, transcriptional co-regulation, similar gene ontology functional annotation) [4,12-14].

An approach to test global *trans *eQTL regions for common biological function is Gene Set Enrichment Analysis (GSEA) [13,14]. GSEA utilizes gene ontology (GO) annotations or other descriptors to define gene sets or gene networks for *a posteriori *tests. Every gene in the transcriptome is ranked relative to the magnitude of its differential expression in response to a treatment. The gene networks are then tested to assess if they demonstrate group responses. Statistical significance is defined by empirical methods where an enrichment score (a rank statistic) is calculated for a randomized data set. After a large number of randomizations, the resulting enrichment scores provide a null distribution from which the critical value for a specified level of significance can be gained. GSEA has been utilized for conducting *a posteriori *tests for non-random association in the network membership of genes controlled by specific QTLs [15]. As one would expect, only those genes with sufficient replication and expression difference provide enough information to allow the identification of eQTLs. Because GSEA is an *a posteriori *test that does not directly use the gene expression value per individual, it is not directly applicable for eQTL mapping. However, GSEA does provide a theoretical foundation for using defined gene networks to analyze eQTLs.

Analysis of variance (ANOVA) is a useful parametric framework that can be used to test networks of genes for global associations. In doing so, a single estimated network expression value for each individual in the mapping population is provided [5,16]. For these applications, ANOVA methods are based on an additive linear model that allows for the partitioning of the sources of variation (e.g., genotype, array, treatment, etc.). Genes are considered nested variables that describe specific *a priori*-defined gene networks (Figure 1). An estimated network expression value can also be calculated by averaging across each individual gene's expression within a network. The use of *a priori *gene network assignment permits the same network to be evaluated and summarized into one value for each individual in a mapping population. For each individual, the resulting single (average) expression value for the network is then used as a quantitative trait in a subsequent QTL analysis that directly identifies eQTLs controlling specific *a priori*-defined gene networks (network eQTLs) (Figure 1 – network averaging approach). This averaging approach is analogous to traditional QTL studies where the average of *n *individuals from the same line or genotype is used to represent the quantitative trait value for that line. Here, the average gene expression from the network represents the phenotype for that network. These networks can be defined *a priori *via GO annotation, or they can be defined in other ways, such as co-regulation observed in other microarray experiments.

We employ ANOVA to analyze microarray data for differential gene network expression among genotypes. We demonstrate that it is possible to identify significant differences in variation at the *a priori*-defined network level between two parental *Arabidopsis thaliana *accessions. We also identify eQTLs associated with networks of genes using a recombinant inbred line (RIL) mapping population derived from the same two parental accessions. Our network averaging approach (Figure 1) for *a priori*-defined network eQTL analysis in the RILs was compared with the summation approach [4,6]. From this investigation we discovered that network members with strong *cis *eQTLs complicate our ability to identify network eQTLs, therefore we explored analytical methods to address this issue. Finally, we discuss candidate transcription factors with *cis*-eQTL that may control a portion of the network eQTLs that are in *trans*, as well as phenotypic traits possibly controlled by the network eQTLs.

## Results

### Network analysis of parental accession variation

**Figure 2:**
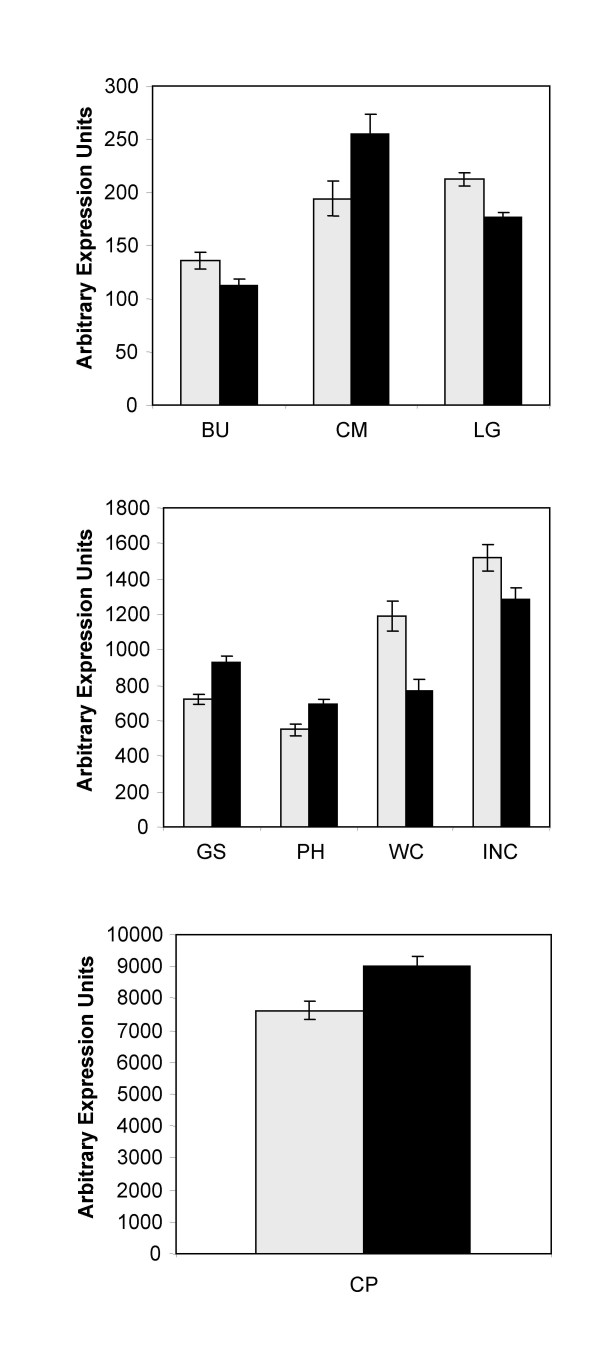
**Network level variation between Bay-0 and Sha**. The eight networks showing significantly differential expression between *Arabidopsis thaliana *accessions Bay-0 and Sha at *P *= 0.05 using Tukey's HSD are illustrated. Network abbreviations are as described in Table 1. Black bars show the average network expression value for Sha while light grey is for Bay-0. Networks not shown did not show a statistically significant difference in the eight Affymetrix ATH1 GeneChips utilized (four Bay-0 and four Sha).

We identified 20 gene expression networks *a priori *(Table 1). A network was defined as an interconnected system of genes. These networks have been shown to be either the transcriptional response to plant/biotic signals or involved in the production of plant defense compounds; the 20 networks include 239 genes. Statistically significant network expression variation between accessions Bay-0 and Shahdara was detected in eight of the 20 networks tested (Figure 2, Table 2 and data not shown). Four networks were expressed at higher levels in Bay-0 and four networks were expressed at higher levels in Sha (Figure 2). An ANOVA with log_2 _normalized expression values showed that the variation due to differential gene expression was approximately the same as that controlled by differential network expression. However, the network × accession interaction was only a small source of variation, about 6% of the gene (network) × accession variation (Table 2).

### Network analysis of glucosinolate gene expression

The well-studied glucosinolate gene network (GS) was used to test the feasibility of an *a priori*-defined gene network approach to map network eQTLs in 148 Bay-0 × Sha RILs, and to compare the network eQTLs to the eQTLs for individual genes. Glucosinolates are metabolites in the Brassicaceae that are believed to control plant responses to insects and pathogens [17]. This a suitable candidate network for testing the network averaging approach since most of the genes in the glucosinolate biosynthetic pathway have been identified and shown to be co-regulated in response to several stimuli [18-20].

**Figure 3:**
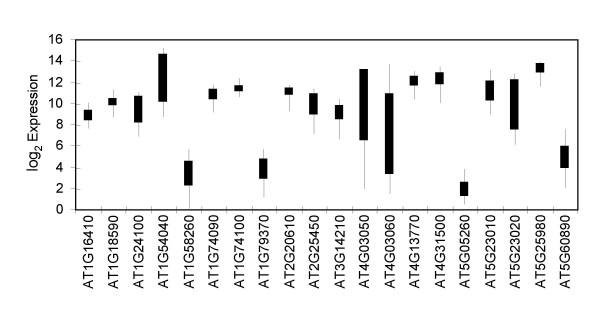
**Variation in gene expression for the Glucosinolate (GS) gene network in Bay-0 × Sha RILs**. Box plot showing the average (+/- one) standard deviation range of log_2 _gene expression in the 148 Bay-0 × Sha RILs for each of the 20 gene members of the GS network. Vertical lines represent the position of the minimal and maximal average gene expression value among the GeneChips corresponding to two biological replicates of each of 148 Bay-0 × Sha RILs.

**Figure 4:**
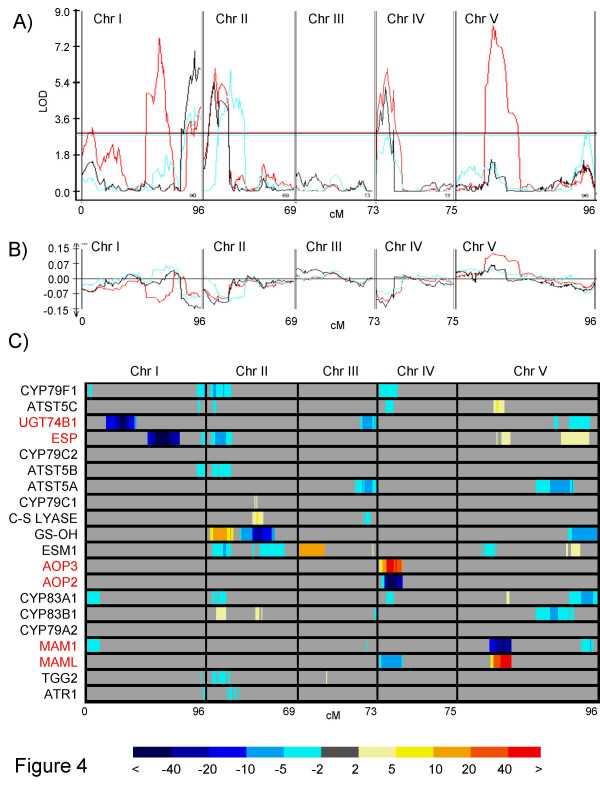
**eQTLs controlling the Glucosinolate (GS) network gene expression**. The vertical lines separate the genome into chromosomes I-V (labeled at the top) with cM progressing left to right along the x axis. The length of each chromosome in cM is indicated at the end of each chromosome. A. Network eQTLs detected (LOD profiles) for the glucosinolate network gene expression estimated as the average of the log_2 _expression across all glucosinolate genes (mean^log2 ^= red profile), average the log_2 _expression across only the glucosinolate genes minus those with a large *cis *effect eQTL (mean^-cis ^= blue profile) and the average normalized expression across all glucosinolate genes (mean^z ^= black profile). The threshold for declaring a QTL significant at α = 0.05 is LOD > 2.8. B. Allele effect trace for the glucosinolate network gene expression estimates shown in A. The y axis is in either log_2 _or z units, depending upon the profile. The effect shown is in terms of the Bay-0 allele. A positive value indicates that the Bay-0 allele has a positive effect on the trait. A negative value indicates that the Bay-0 allele has a negative effect while the Sha allele has a positive effect on the trait. C. eQTLs controlling the log_2 _expression of the individual genes in the glucosinolate network. The color schematic at the bottom shows the LOD score scale with the directionality of effect indicated by the color. Only significant QTLs are shown. An eQTL for which the Bay-0 allele has a positive effect is shown by differing shades of yellow and red, and darker intensity indicates a greater LOD score at that genetic position. An eQTL where the Bay-0 allele has a negative effect is shown by differing shades of blue, again with darker intensity indicating a greater LOD score. Gene names in red indicate those with a *cis*-eQTL that controls > 50% of the phenotypic variation for that gene.

The GS network's expression value per RIL was determined using the mean log_2 _expression across the 20 GS genes in each RIL (mean^log2^). A large difference in the average log_2 _expression values for individual genes was evident; the more highly expressed genes contributed a greater proportion to the mean network expression value (Figure 3). The mean^log2 ^for the GS gene network identified five network eQTLs (Figure 4A). For four of these network eQTLs, the Bay-0 allele had a negative effect on the network's expression value, while for one network eQTL the Bay-0 allele had a positive effect (Figure 4B).

Previous studies have shown that *cis *expression polymorphisms control glucosinolate gene activity and/or expression in other mapping populations [21-23]. Genes with predominant *cis *effects have the potential to contribute disproportionately to the overall expression variation of a network. Therefore, we expect this *cis *effect when estimating the GS network expression variation. We mapped eQTLs controlling each of the 20 GS genes (Figure 4C) and identified six transcripts where a *cis *eQTL controlled > 50% of the phenotypic variation (*UGT74B1*, *ESP*, *AOP3*, *AOP2*, *MAM1 *and *MAML*) (Figure 4C). All except the *UGT74B1 *transcript was previously known to have *cis*-controlled expression variation [21-24]. The *cis *eQTL for *ESP*, *AOP3*, *AOP2*, *MAM1 *and *MAML *overlapped with three network eQTLs identified using the mean^log2 ^network expression estimate, suggesting that the *cis*-eQTL for these highly expressed genes may be generating network eQTL with this network expression average.

Large *cis*-eQTLs would likely mask the network level control on an individual gene's expression due to the *cis *polymorphism's high level of expression variation, thus dominating the network average and obscuring the *trans *effects. To remove the impact of large *cis*-eQTLs on the GS gene expression network, we estimated the GS network log_2 _mean expression value based on a filtered dataset that eliminated the six genes exhibiting large *cis *effect eQTL (mean^-cis^). A comparison of mean to median estimates in the presence and absence of the *cis *affected genes showed that removing them from the pathway brought the two measures in closer alignment (data not shown). A comparison of eQTLs detected with mean^log2 ^versus mean^-cis ^showed that two of the network eQTLs, in the middle of chromosomes I and V, disappeared; these were likely due solely to the effect of the *ESP*, *MAM1 *and *MAML cis*-eQTLs (Figure 4). In contrast, three other network eQTLs were reproducible. The network eQTL at the top of chromosome II had a slightly shifted position. Without cloning of the underlying polymorphism, it is not possible to determine if this is a significant change in peak position or due to the use of an altered gene list for the network. An additional small network eQTL was identified with mean^-cis ^at the bottom of chromosome V (Figure 4).

A wide range of log_2 _expression values for individual genes in a network (Figure 3) may also affect network eQTL identification. To evaluate the effect of rescaling gene expression data, the mean expression value for the GS network was estimated using the standardized z-values for all 20 genes in each RIL (mean^z^). The mean and median for the pathway values were very similar for z-scaled gene expression values (data not shown). An analysis of the GS network based on the mean^z ^estimate identified nearly all the same network eQTLs as the mean^log2 ^estimate, except that the putative *cis*-eQTL from *ESP*, *MAM1 *and *MAML *disappeared (Figure 4A). As expected, the network eQTL plots generated in the absence of the six genes that dominated the network (mean^-cis^) were comparable to the results based on the mean^z ^estimates since the normalization procedure scaled the six genes exhibiting large effects (Figure 4A).

### Network eQTL analysis

**Figure 5:**
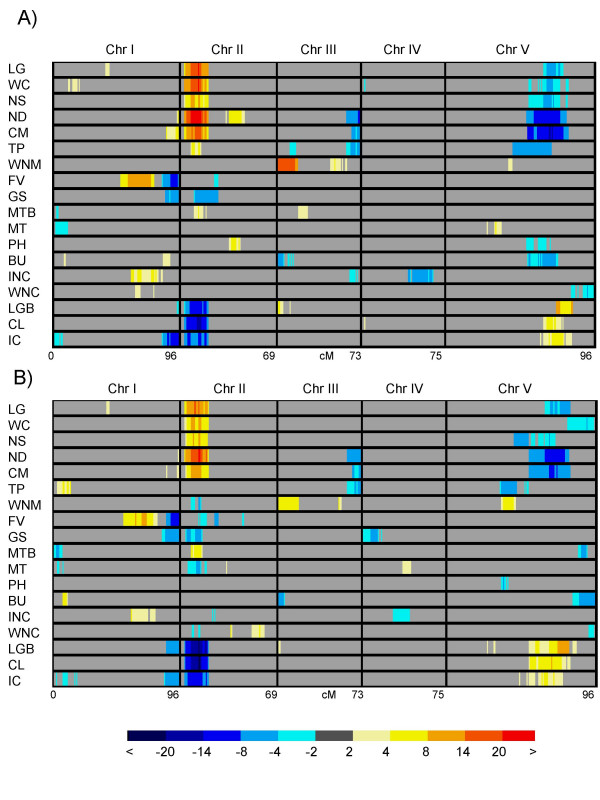
**Network eQTL controlling *a priori *defined gene expression networks**. The vertical lines separate the genome into chromosomes I-V (labeled at the top) with cM progressing left to right along the x axis, and the length of each chromosome in cM is at the end of each chromosome. The color schematic shows the LOD plot for each gene with the directionality of effect indicated by the color. Only significant QTLs are shown. A network eQTL where the Bay-0 allele has a positive effect is shown by differing shades of yellow and red, and darker intensity indicates a greater LOD score at that genetic position. A QTL where the Bay-0 allele has a negative effect is shown by differing shades of blue, again with darker intensity indicating a greater LOD score. The networks are ordered using median centered Spearman rank clustering of the effect traces along the genome; as a result, networks showing similar network eQTLs and effect directionality are located adjacent to each other. A. Network eQTL for gene expression networks obtained using mean^-cis^. B. Network eQTL for gene expression networks obtained using mean^z^.

We implemented the *a priori *network eQTL approach to identify network eQTLs for all 20 *a priori *defined gene expression networks (Table 1) used for the comparison of the Bay-0 and Sha parental accessions. Statistically significant network eQTLs were detected in the 148 Bay-0 × Sha RILs for 18 out of 20 networks (Figure 5). The analyses based on the mean^-cis ^and mean^z ^estimates generated very similar network eQTL plots for each of the 18 networks (Figure 5A and 5B). Multiple networks identified the network eQTLs at the top of chromosome II and the bottom of chromosome V, suggesting that these regions contain large global-effect network eQTLs, but these regions did not affect all expression networks in the same way. The presence of the Bay-0 allele at the network eQTL on the top of chromosome II up-regulated the LG, WC, NS, ND, CM, TP and MTB networks, while the same allele down-regulated the GS, MT, WNM, LGB, CL and IC networks (Figure 5). Interestingly, the Bay-0 allele at the network eQTL on the bottom of chromosome V had the reverse effect on these same networks. Network eQTLs located at the bottom of chromosome I and III also affected multiple gene expression networks, albeit not as many as the aforementioned regions. While most network eQTLs appeared to be associated with multiple networks, there were network eQTLs associated with only a single network, such as the network eQTL on chromosome I for the LG network.

To determine if physical clustering of genes in the same network affected the identification of network eQTLs, we plotted the genomic position of each gene within a network and compared it to the position of the network eQTLs detected with the mean^z ^expression values (data not shown). In the LG network, 20% of the genes are present in a single tandemly duplicated gene cluster on the top of chromosome I that co-localizes with a small effect network eQTL, suggesting that this network eQTL may be due to the sum of small *cis*-acting QTLs (data not shown). However, within our collection of 20 networks and 239 genes, there was no other instance of genomic clustering of network genes. Thus, the vast majority of network eQTLs cannot be explained by the additive effects of small *cis*-effect eQTLs for tandemly duplicated genes, and are likely *trans*-acting network eQTLs controlling the specific network in question.

### Summation analysis of eQTLs

**Figure 6:**
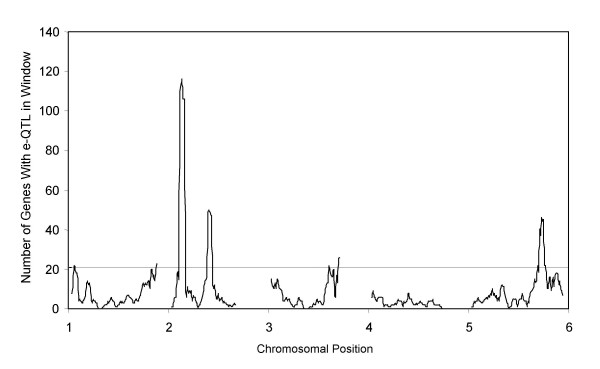
**Broad effect eQTL regions detected via the summation approach**. The number of genes showing eQTLs within a 5 cM sliding window is shown. The *P *= 0.05 cutoff for significant enhancement (horizontal line) is equal to 27 genes with an eQTL within a 5 cM sliding window. A total of 245 genes were used for mapping eQTLs. The five chromosomes lie along the x axis and begin at the corresponding number such that 1 is the start of chromosome I, etc. All five chromosomes are < 100 cM in this sample of 148 RILs of the Bay-0 × Sha population.

One approach to identifying *trans*-eQTLs that affect the expression of multiple genes is to identify the eQTLs for each individual gene and conduct a sliding window, or binning analysis, that scans the genome and adds together (i.e., sums) the number of eQTLs in each region [4,12]. Permutation thresholds provide the ability to identify genomic regions that contain a significant enrichment for the number of genes with an eQTL in that region (i.e., an eQTL 'hotspot'). We identified network eQTLs using the summation approach and compared the results to those identified by our *a priori *network averaging approach (Figure 1). We used the 705 eQTLs controlling the expression of the 245 genes in this study (239 genes in 20 networks, plus six transcription factors affecting the FV network; Table 1). This yielded an average of 2.9 eQTLs per gene (ranging from 0 to 7 eQTLs), with only 15 genes having no identifiable eQTL (Additional file 2). Thirty-eight of the 245 genes had a *cis*-eQTL controlling > 50% of the phenotypic variance. The eQTLs for all of the genes were then used for the summation approach to global eQTL analysis (Figure 1) [6]. Three genomic regions, on the top and middle of chromosome II and the bottom of chromosome V, showed a significant enhancement in eQTL density above the 0.05 significance threshold (Figure 6). The top of chromosome II and the bottom of chromosome V were also associated with network eQTLs using the mean^z ^and mean^-cis ^analysis (Figure 5 and 6). Three other regions, the top and bottom of chromosome I and the bottom of chromosome III, were suggestive in that they were barely above the 0.05 significance threshold (Figure 6).

### Phenotypic QTL clustering

**Figure 7:**
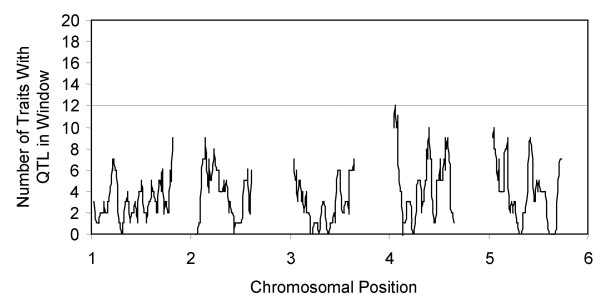
**Summation approach to phenotype QTL clustering**. The number of physiological traits showing a QTL within a 5 cM sliding window is shown. One thousand permutations of this data showed that the *P *= 0.05 threshold for significant enhancement is 12 traits with a QTL per sliding window (horizontal line). QTLs for 62 phenotypic traits were used for this analysis. The five chromosomes begin at the corresponding number such that 1 is the start of chromosome I, etc.

Genomic regions containing *trans*-acting network eQTLs may be more likely to control phenotypic trait variation. We combined 62 physiological, biochemical and morphological trait QTLs detected in other studies of the Bay-0 × Sha RIL population, and conducted the summation approach to search for genomic hotspots associated with an enriched number of phenotypic QTLs. Based on a permutation threshold, no statistically significant grouping of phenotypic QTLs was identified (Figure 7). It is possible that these particular phenotypic traits are not controlled by the network eQTLs identified in our study.

### Candidate transcription factor identification

**Figure 8:**
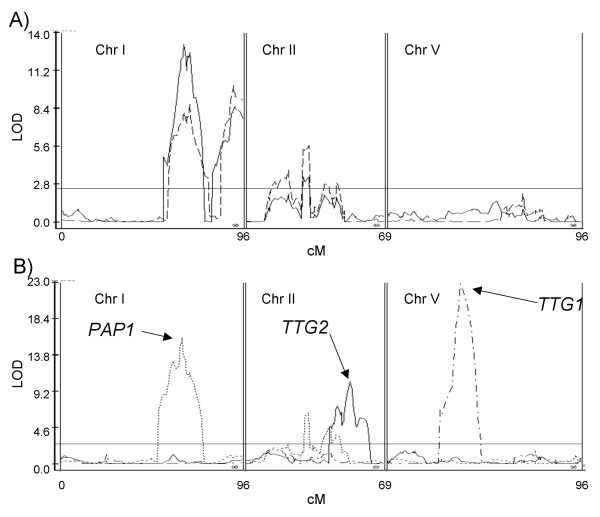
**Flavonol (FV) biosynthetic gene expression network eQTLs versus transcription factor eQTLs**. The vertical lines separate the genome into chromosomes I-V (as labeled at the top) with cM progressing left to right along the x axis and the length of each chromosome in cM at the end of each chromosome. Only chromosomes I, II and V were included in the figure since chromosomes III and IV did not contain eQTLs for any of the traits described. A) Network eQTLs controlling the expression of flavonol biosynthetic genes. The solid black line is the mean FV network expression utilizing the log^2 ^normalized gene expression. The dashed black line is the mean network expression utilizing the z score gene expression. B) eQTLs controlling the expression of transcription factors known to regulate the flavonol biosynthetic gene expression. *TTG1 *(AT5G24520), *TTG2 *(AT2G37260) and *PAP1 *(AT1G56650) detected eQTLs, while *TT2 *(AT5G35550), *TT16 *(AT5G23260) and *TT8 *(AT4G09820) did not. The arrows show the genomic position of the *TTG1*, *TTG2 *and *PAP1 *genes.

*Trans*-acting network eQTLs are hypothesized to be regulated by transcription factor (TF) variation. To determine if we could identify TFs with *cis*-acting eQTLs that may control the *trans*-acting network eQTLs, we used the FV (flavonol) gene expression network since it is transcriptionally regulated by at least six transcription factors [25-28]. Mapping of eQTLs for six TFs known to control the FV network showed that three TFs, *PAP1*, *TTG1 *and *TTG2*, were associated with large effect *cis*-eQTLs (Figure 8). Of these, only the *PAP1 cis*-eQTL co-localized with a FV network eQTL (Figure 8). Interestingly, *PAP1 *also showed *trans*-acting eQTL in the same regions of chromosome II as the FV network. Thus, *a priori *information can be used to identify potential candidate transcription factor variation controlling expression networks. Furthermore, the *a priori *information was helpful for only one of the FV network eQTLs, suggesting that studying natural variation for polymorphic gene expression patterns is likely to identify unknown factors regulating expression networks.

## Discussion

The use of the network averaging approach based on *a priori *defined gene networks was effective in identifying network eQTLs (Figures 1, 4 and 5). We located a number of *trans*-acting network eQTLs that controlled the expression of varying numbers of gene networks (Figures 4 and 5). These network eQTLs were detected in similar genomic regions as those identified via the summation eQTL approach (Figures 5 versus 6). Thus, the use of *a priori *networks in combination with an efficient statistical analysis can serve to identify *trans*-acting regulatory eQTLs.

The use of *a priori *networks in conjunction with an ANOVA successfully identified network expression differences between two parental accessions, and indicated that about half of the 20 networks considered exhibited basal expression level differences between Bay-0 and Sha. These differences were split equally between Bay-0 and Sha. Since these networks represent groups of genes, these results suggest that there is genetic variation that coordinately impacts the expression of these genes. The presence of network-level variation affects comparisons of the expression of specific genes across accessions such that if gene expression networks are differentially expressed between two accessions, then the genes within those networks will be subjected to different regulatory patterns. In this case, understanding network-level variation among genotypes will help elucidate how individual genes respond to a signal in comparison to their network response.

Because the network averaging approach depends on *a priori *network information, it is only as robust as the defined gene networks. If the prior experiments were not properly designed, replicated, conducted and analyzed, then the networks defined by those experiments will not be reliable nor yield biologically meaningful eQTL results. Biological sources of error are relevant to the underlying assumption that the variation utilized to generate the *a priori *networks is related to the variation being studied in the new experiment. For example, the use of *a priori*-defined networks identified by comparing gene expression in different tissues may not be appropriate for querying a new experiment focused on a single tissue's response to pathogen attack. Instead, the use of networks defined by responses of a similar tissue challenged by a different pathogen would be more appropriate for *a priori *network analysis.

### Comparison of global eQTL approaches

We compared the results from an analysis based on the summation approach [4,6] with the results from our network averaging approach analyses (Figure 1) using mean^log2^, mean^-cis ^and mean^z ^for *a priori*-defined networks. Both approaches identified two genetic regions with broad effects on gene expression, located on top of chromosome II and the bottom of chromosome V (Figures 5 and 6). The *a priori*-defined network approach also identified several regions that were detected as suggestive in that they were just above the permutation threshold for significance based on the summation approach. Our comparison suggests that the summation approach is likely biased towards detecting global eQTLs that have broad impacts on gene expression. In contrast, network averaging allows sub-classification of the genes prior to eQTL analysis, which can help identify underlying patterns associated with the global view obtained by the summation approach. In addition, an analysis based on network averaging with mean^-cis ^and mean^z ^estimates can be used to remove or reduce the effect of large *cis*-acting genes to reveal *trans*-acting eQTLs and identify global regulatory eQTLs. Therefore, our results suggest that the summation and network averaging approaches should be viewed as complementary methods that in combination provide a more complete assessment of the genetic architecture of global transcriptome variation and the underlying complexity of gene networks.

### Transgressive expression variation

Twenty networks containing 239 genes identified several broad-effect network eQTLs in the Bay-0 × Sha RIL population. Interestingly, two of these network eQTLs (at the top of chromosome II and the bottom of chromosome V) show opposite allelic effects both with respect to each other and with respect to their impacts on the networks. The opposite allelic effects observed for these two network eQTLs suggests that this RIL population has transgressive segregation affecting gene expression network values such that Bay-0 has a +/- allele combination and Sha has a -/+ combination which negate each other. This agrees well with the relatively small differences we detected in gene network expression for the Bay-0 and Sha parental accessions. In contrast, some of the RILs have recombinant (non-parental) +/+ and -/- allele combinations at these network eQTLs, which is not unexpected in a segregating population.

### Network eQTLs versus phenotypic trait QTLs

We did not observe a significant enrichment of phenotypic QTLs within regions showing broad-effect network eQTLs (Figures 5, 6 and 7). This lack of association suggests that these network eQTL regions do not have detectable effects on these particular traits. However, two shoot growth QTLs (*DM10.3 *and *DM10.8*) [29] with opposite allelic effects co-localize with our two main network eQTLs, suggesting that it may be more informative to examine network specific-eQTLs and the phenotypic consequences expected from the predicted biological function of the networks. The use of *a priori*-defined gene expression networks allows generation of specific hypotheses about the phenotypic effect of network eQTLs. The GS and CL networks are known to be repressed by oxidative stress and salicylic acid, while the NS, ND, CM and TP networks are known to be induced by these two factors, suggesting that the network eQTLs associated with these networks may involve some facet of oxidative stress and/or salicylic acid homeostasis within the RIL population. Validating this hypothesis and determining network associations will require the cloning and characterization of the underlying genetic polymorphisms.

### Network-specific eQTLs

Our predominant interests in Arabidopsis involve plant/pathogen and plant/pest interactions which are controlled by a variety of characterized transcriptional and metabolic response networks [17,30-33]. The NS, ND, CM and TP networks are involved in plant/pathogen responses and are known to be co-regulated in response to biotic attack (See Table 1 for references). This agrees well with our observation that these networks are typically associated with similar genomic locations of network eQTLs (Figures 4 and 5). Interestingly, one network eQTL at the bottom of chromosome III is specific to these networks, suggesting that it may specifically influence variation in plant/pathogen interactions in this population.

The FV, GS and IC networks are induced by both methyl jasmonate (MeJA) and insect herbivory. While these networks did not show complete agreement in the location of their network eQTLs, they were all affected by a network eQTL at the bottom of chromosome I which appeared to be specific for these three networks. Interestingly, this region also influenced the ND and CM networks, albeit with opposite parental allele effects on the FV, GS and IC networks. The ND and CM networks are known to be MeJA-repressed. Thus, the observed opposite parental allele effects on MeJA-inducible and MeJA-repressible gene networks suggests that this region may be controlling MeJA homeostasis and/or signaling.

The bottom regions of chromosomes I and III exhibited a slightly significant enrichment for eQTLs using the summation approach. This slight significance would typically lead to these QTLs being noted but not necessarily pursued. However, the *a priori *network averaging analysis suggested that these regions may be specific for defined regulatory networks involved in plant/biotic pest interactions. This defined regulatory role will limit the number of transcripts that can be controlled by variation in these regions and minimize the ability to detect an eQTL enrichment signal on a global regulatory analysis with the summation approach. Thus, by utilizing *a priori *information to sub-categorize the global gene expression network, it is possible to identify network-specific eQTLs that might otherwise be overlooked.

## Conclusion

The *a priori *network averaging approach uses a novel statistic to summarize each individual's (defined) network in the mapping population. The actual statistical analyses are based on well-established statistical methods for identifying QTLs. In this application, the identified QTLs are controlling genes sharing a common biological function, and thus are referred to as network eQTLs. This approach has the major advantage of allowing researchers to apply their biological knowledge of gene associations from previous work to the analysis of network eQTLs. The *a priori *network averaging approach is also complementary to other methods, such as the summation approach and GSEA, and as presented here provides a framework within which transcriptome data can be analyzed for the purpose of addressing hypothesis-driven questions. For example, one could define the networks based on genes expressed in specific cell types (e.g., trichomes versus stomates) to identify eQTLs potentially controlling the development of these tissues. Since it is likely that each gene is a member of multiple semi-independent networks, grouping genes into multiple different networks with subsequent eQTL analysis may enhance our understanding of how gene networks interact and control phenotypic outputs.

Two major questions that remain to be addressed are: what is the relationship between the quantitative trait phenotype and gene expression values, and what is the relationship between phenotypic trait QTLs and eQTLs? Using *a priori *network definitions, it may be possible to directly identify network eQTLs for defined metabolic pathways or trait phenotypes such as pathogen resistance. Thus, by mapping QTLs for the specific metabolites and/or pathogen resistance in the same population under the same conditions that are used for global gene expression analysis, it may be possible to directly compare the genetic architecture of eQTLs controlling the transcripts to the resulting measurable phenotype. The use of *a priori *definitions for network eQTL identification has enormous potential for providing direction toward future transcriptomics eQTL analyses, as it facilitates a direct test of the relationship between phenotypic trait and transcript genetic architecture.

## Methods

### Plant material and experimental conditions

Seeds for *Arabidopsis thaliana *accessions Bayreuth (Bay-0), Shahdara (Sha), and a Bay-0 × Sha recombinant inbred line (RIL) population [34] were obtained from TAIR (stock #CS57920)[35]. The RIL F_8 _plants and parental accessions were grown in a growth chamber at UC-Davis, allowed to self-pollinate, and seed was harvested from individual plants to produce sufficient seed for each homozygous F_9 _line for our replicated experiments.

Replicated factorial experiments were conducted with Bay-0 and Sha plants grown on three separate dates in a growth chamber at UC-Davis under short day conditions (8 hr light at 100–120 μEi, 20°C day/20°C night) to delay flowering and maintain plants in the vegetative phase. At six weeks post-germination these plants were sprayed to run-off with 0.02% Silwet L77, a surfactant (Lehle Seeds, Round Rock, TX, USA)[36] as the control treatment for a larger factorial experiment (to be described elsewhere). All rosette leaves of three plants per genotype-time point combination were bulk harvested 4, 28, or 52 hrs post-Silwet-treatment and quick-frozen in liquid nitrogen.

Subsequently, the Bay-0 × Sha RIL experiment was conducted, during which five plants per biological replicate for each of 148 RILs, plus parental controls Bay-0 and Sha, were grown in growth chambers under identical short day conditions (8 hr light at 100–120 μEi, 20°C day/20°C night) for six weeks. At six weeks post-germination, the plants were sprayed to run-off with 0.02% Silwet as the control treatment for a larger experiment involving salicylic acid response (to be described elsewhere); plants were harvested 28 hours post treatment. All rosette leaves from three plants per genotype were bulk harvested and quick-frozen in liquid nitrogen. Due to limitations in growth chamber space, the two biological replications of 148 RILs plus parental controls were grown sequentially, one complete replication at a time.

### RNA isolation and microarray hybridization

Total RNA was extracted with TRIzol (Invitrogen, Carlsbad, CA, USA), purified on RNeasy columns (Qiagen, Valencia, CA, USA), then used as a template for cDNA synthesis, as recommended by the GeneChip manufacturer (Affymetrix, Santa Clara, CA, USA, ). Biotinylated cRNA was synthesized, and hybridized according to the manufacturer's guidelines to Affymetrix ATH1 GeneChips representing 22,810 *A. thaliana genes*. GeneChips were scanned on an Affymetrix GeneArray Scanner using GCOS software (Affymetrix, Santa Clara, CA, USA). In total, two sets of Gene Chip data from independent experiments were generated: Bay-0 and Sha parental data from the factorial (time point × accession) experiment, and a RIL data set consisting of two biological replicates of 148 RILs plus Bay-0 and Sha parental controls. The microarray data used for this study is available at ArrayExpress under accession numbers E-TABM-61 and E-TABM-62 and at elp.ucdavis.edu.

In order to allow comparisons of gene expression values across GeneChips, global scaling was used to adjust the trimmed mean signal of each GeneChip probe array to a target signal value of 600 (Affymetrix GeneChip Operating Software User's Guide, Version 1.3, ). Scaled gene expression values were obtained for all probe sets for each GeneChip and used for all subsequent data analyses. We did not remove genes that contained single feature polymorphisms (SFPs). Previous work has shown that one or a few SFPs per gene does not lead to a significant change in the overall gene expression estimate [37].

### Microarray quality control

The scanned image of each GeneChip was visually inspected for artifacts, and routine quality control parameters were checked in accordance with the manufacturer's recommendations (GeneChip Expression Analysis Data Analysis Fundamentals P/N 701190)[38]. In addition, the parental and RIL assignment for each GeneChip was confirmed by examining the expression levels of 192 genes identified as diagnostic, and then clustering the microarrays based on genotype to ensure that biological replicates per genotype clustered together. The biological replicates for each of the 148 RILs were appropriately clustered. In addition, the RIL haplotypes obtained from SFPs scored as markers from these same GeneChips [39] were consistent with those determined previously by microsatellite analysis of genomic DNA (Loudet et al. 2002).

### *A priori*-defined networks

Gene expression networks listed in Table 1 were identified using multiple sources of information. Published studies using ATH1 GeneChips were used to group genes into networks based on coordinated expression [19,31,32] to identify the FV, GS, WC, WNM, WNC, IC, INC, ND, and NS networks (Table 1). Unpublished experiments focused on the rapid (12 hour) induction of gene expression in response to *Botrytis cinerea *infections were utilized to define members of the BD and BU networks (Kliebenstein *et al*. unpublished data). Published studies on multiple biosynthetic pathways in Arabidopsis showing coordinate regulation of genes in response to a variety of stimuli [19,40,41] were used to define the putative gene expression networks CM, CL, FV, GS and TP (Table 1). To test the relationship between biosynthetic pathway membership and coordinated gene expression, we identified an additional set of biosynthetic pathways and their genes to delimit putative gene expression networks LG, LGB, MT, MTB, PH, and SN [42,43]. The lignin and methionine biosynthetic pathways were further divided into two sub-networks (LG and LGB; MT and MTB) based on which genes were either positively or negatively correlated with the CL network (*P *< 0.05 using Tukey's HSD test). The gene members of each network are listed in Additional file 1. FTF includes six known transcription factors that may regulate the FT network. FTF is not defined as an actual gene expression network *per se*; it is included in our study to permit an analysis of co-localization of transcription factors and *trans*-acting network eQTLs.

To test the similarity of gene co-expression within these networks (Table 1) in other datasets, we obtained transcript expression values for all network genes from 2,391 publicly available ATH1 GeneChips (affymetrix.arabidopsis.info) and conducted a Pearson correlation analysis of all pairwise combinations of genes using Excel. This microarray dataset represents a broad sampling of treatments, growth conditions, developmental stages, tissues and genotypes (affymetrix.arabidopsis.info). The average Pearson correlation of transcript expression values for genes within the same network was *r *= 0.228 compared to an average of *r *= 0.016 for random pairs of genes from different networks. The higher *r *value for genes within the same network suggests that they are likely to be co-expressed, supporting our use of the network definitions listed in Table 1.

### Bay-0 versus Shahdara parental network analysis

Gene expression values from multiple ATH1 GeneChips for the parental accessions, Bay-0 and Sha (four per accession) were used to test differential expression at the network level using *a priori *network definitions. To simplify the analysis, we only utilized the 28 hour post-Silwet treatment GeneChips.

A mixed linear model ANOVA was used to analyze the log_2 _gene expression values. This model partitioned the sources of variation (e.g., genotype, network, gene, replicate and their various interaction terms) to improve accuracy and enhance experimental interpretation of differential expression [5]. Statistically significant gene network expression differences between Bay-0 and Sha were tested using a split-plot mixed linear model with a random array effect. The expression level of gene *k *from network *j*, measured from the parental accession *i *for the chip replication *r *is denoted as *y*_*ijkr*_. The ANOVA model for the log-transformed expression is:

*log*_2_(*y*_*ijkr*_) = *μ *+ *P*_*i *_+ *N*_*j *_+ *G(N)*_*jk *_+ *R*_*r *_+ *PNij *+ *PG(N)*_*ijk *_+ *PR*_*ir *_+ *NR*_*jr *_+ *RG(N)*_*rjk *_+ *PRN*_*ijr *_+ *ε*_*ijkr*_

where *i *= 1, 2, *j *= 1, ...,20, *k *= 1, ..., 239 (the six flavonol TFs were not included in this analysis as they are not a gene network) and *r *= 1, 2. The main effects are denoted as *P*, *N*, *G *and *R *and represent parental accession, *a priori *defined network, gene, and replication, respectively. The sub-plot error, *ε*_*ijkgr*_, is assumed to be normally distributed with mean 0 and variance σ_ε_^2^. Average network expression values were estimated for each accession utilizing SAS version 9.1 with the above ANOVA model (SAS Institute, Cary, NC, USA). Significant network expression differences between Bay-0 and Sha were tested at α = 0.05 using the mean network expression levels and the type I family-wise error controlled utilizing Tukey's HSD test.

### Network averaging approach to eQTL identification

We investigated three different approaches for estimating the average network expression value for each RIL to identify network eQTLs (Figure 1 – network averaging approach). The first approach used the log_2 _of each gene's expression value obtained from the GeneChip. The log transformed data were approximately normally distributed with decreased magnitude differences between the highest and lowest expressed genes within a network. The expression values for all genes within a network were used to estimate the network mean expression value individually for each RIL, and are denoted as mean_i _^log2 ^(i = 1,...,148). Our second approach estimated the network mean expression value in each RIL after eliminating all genes with a *cis*-eQTL that accounted for most of that gene's expression variation. Genes with a *cis *polymorphism that cause large variation in expression between alleles inordinately skew the network's mean expression value and limit the identification of *trans*-acting network eQTLs. These *cis*-influenced genes, identified as described in a following section, were removed and then the network mean expression value (mean_i_^-cis^) was estimated for each individual (i = 1,...,148) using the log_2 _expression of the remaining genes within each network. Our third approach employed a standard normal (z) distribution, N(0,1), to standardize each gene's expression across the RILs. The expression value for each gene for each RIL was transformed to the corresponding z score by subtracting the average and dividing by the standard deviation (i.e., using the standard function in Excel). All genes were included and the standardized values for all genes within a network were then used to calculate a network mean expression value (mean_i _^*z*^) for all i = 1,...,148. In order to understand and evaluate the benefits of each network averaging approach (Figure 1), all three estimates (mean^log2^, mean^-cis ^and mean^z^) were used as unique quantitative traits in the subsequent network eQTL analyses. All averaging approaches assume that the expression across the RILs for the different genes exhibit a linear and independent relationship among genes within a network.

### Network eQTL mapping

A high-density SFP-based marker linkage map for the 148 Bay-0 × Sha RILs was obtained [39] and used to map network eQTLs for the network averaging estimates (mean^log2^, mean^-cis ^and mean^z^). Furthermore, within the *a priori*-defined network, individual gene log_2 _expression values for each RIL were used to map eQTLs. All data were analyzed with QTL Cartographer v3.0 [1,44]. Composite interval mapping was conducted using Zmap (Model 6) with a 10 cM window and an interval mapping increment of 2 cM. Forward regression was used to identify five cofactors per gene (quantitative trait). The declaration of statistically significant eQTL is based on permutation-derived empirical thresholds. One thousand permutations were employed for each gene (quantitative trait) [45,46]. To summarize and display eQTLs for individual genes and networks, TKlife was used to generate heat plots [47].

### Identification of *cis*-effect for individual gene eQTLs

Log_2 _expression values for each transcript for 245 genes (which includes the six flavonol TFs) were employed to map gene-specific eQTLs in the RILs. The flavonol TFs are included for a later analysis of transcription factor co-localization with *trans *network eQTLs for the FV network. This analysis identified 705 eQTLs, and QTL Cartographer was used to estimate the percent phenotypic effect of each eQTL for each gene. The results were then sorted to identify genes that had a *cis*-localized eQTL. Any eQTL located within 2 cM of the genomic position of the gene encoding the transcript was identified as a *cis*-localized eQTL. The genes with a *cis*-eQTL controlling > 50% of the gene's expression variation were classified as genes with major *cis*-acting eQTL. These genes were not included in the mean^-cis ^method to estimate network expression.

### Sliding window analysis of QTL position for individual gene eQTLs

To identify genomic regions containing a greater number of eQTLs than expected by chance, we conducted a sliding window analysis (Figure 1 – Summation Approach). The genetic positions of all 705 eQTLs identified for the 245 genes were estimated with QTL Cartographer, and the number of eQTLs per chromosome was determined within a 5 cM sliding window, starting at the top of each chromosome and progressing down the chromosome in 1 cM steps.

To estimate the threshold limit at significance level of 0.05 for the frequency of genes with an eQTL within a 5 cM sliding window, the positions of the 705 eQTLs were permuted across the genome 1000 times. The sliding window analysis was repeated for each permutation, and the maximum number of eQTLs per sliding window per permutation was obtained. Using the distribution of the maximum number of eQTLs, the 0.05 threshold provides the criterion for declaration of significant results (21 eQTLs/5 cM window). The bounds on this empirical distribution were 27 and 15 eQTLs/5 cM window, respectively.

### Sliding window analysis of QTL positions for phenotypic traits

To investigate whether genomic regions containing more phenotypic QTLs per region than expected by chance are associated with the network eQTLs, we conducted a sliding window analysis and compared the results (Figure 1 – Summation Approach, using Phenotypic QTL instead of eQTL). A diverse range of 62 biochemical, morphological and plant/biotic interactions traits were included (Additional file 3) [29,30,34,48-52]. The 62 traits identified 281 phenotypic QTLs based on the 38 microsatellite marker map for 411 Bay-0 × Sha RILs [34], resulting in an average of 4.5 QTLs per trait. Because these data were measured on all 411 RILs, we used the highest-resolution map available for this RIL collection. The empirical threshold for a significance level of 0.05 for the frequency of traits with a phenotypic QTL per 5 cM sliding window was estimated as described above.

## Authors' contributions

DJK originated the summary measures mean^log2^, mean^-cis ^and mean^z ^for use in the QTL analysis. He conducted the statistical and QTL analyses, provided intellectual insight into development of the statistical methodologies described in conjunction with writing the manuscript.

MALW managed and conducted the microarray experiments and aided in reviewing and editing the manuscript.

HVL aided in translation of the microarray data files, assisted in data file management, and aided in reviewing and editing the manuscript.

OL provided phenotypic trait QTL data for comparison to the eQTL results and aided in reviewing and editing the manuscript.

RWD provided intellectual insight into the design of the microarray experiments, and aided in writing, reviewing and editing the manuscript.

DAS initiated, designed and managed the microarray experiments, provided intellectual insight into development of the statistical methodologies described and aided in writing, reviewing and editing the manuscript

## Supplementary Material

Additional File 1 **Gene members of the gene expression networks**. ^a^Network indicates the network that the gene is a member of. ^b^Gene is the Atg nomenclature for each gene listed. ^c ^> 50% *Cis *QTL indicates whether the gene has a *cis*-eQTL controlling greater than 50% of the expression phenotypic difference. Click here for file

Additional File 2 **eQTL positions for all individual genes in this study**. ^a^Network indicates the network that the gene is a member of. ^b^Gene is the Atg gene nomenclature for each gene listed. ^c^eQTL position indicates the chromosome (I, II, III, IV or V) and the cM of the eQTL LOD peak on that chromosome, separated by a period. The position of all significant eQTLs for each gene is shown. ^d^eQTL # is the number of eQTLs identified for each individual gene. Click here for file

Additional File 3 **Traits used for QTL analysis in this study**. ^a^QTL Name is the abbreviation utilized for that trait. ^b^Trait is the phenotype measured to identify the associated QTLs. ^c^Reference is the literature reference in which the trait is described. Unpublished QTL data are either from the Loudet or Kliebenstein laboratories. Click here for file

## Figures and Tables

**Table 1:** *A Priori*-Defined Gene Networks.

Abbreviation^a^	Network Biological Description	# Genes^b^	# *Cis *QTL^c^	Reference^d^
BD	Genes down regulated by Botrytis	6	0	Unpublished (Kliebenstein)
BU	Genes up regulated by Botrytis	10	0	Unpublished (Kliebenstein)
CM	Camalexin biosynthetic pathway	7	1	[55]
CL	Genes involved in Photosynthesis	27	1	ABRC
FV	Flavonoid biosynthetic pathway	15	1	[19, 56]
FVTF^e^	Transcription Factors for FV	6	3	[57]
GS	Glucosinolate biosynthetic pathway	20	6	[18, 19]
LG	Lignin Production	43	11	[58] and this work
LGB	Lignin Production	9	1	[58] and this work
MT	Methionine biosynthetic pathway	5	0	ABRC and this work
MTB	Methionine biosynthetic pathway	5	1	ABRC and this work
PH	Phenylalanine biosynthetic pathway	11	5	ABRC
SN	Sinapate biosynthetic pathway	3	0	[59]
TP	Tryptophan biosynthetic pathway	9	2	[60]
WC	Wound-inducible genes controlled by COI1	7	1	[33] Net. C
WNM	Wound-inducible genes, no MeJA	4	0	[33] Net. G
WNC	Wound-inducible genes, not controlled by COI1	8	1	[33] Net. D
IC	Insect-inducible genes controlled by COI1	14	1	[33] Net. A
INC	Insect-inducible genes, not controlled by COI1	13	0	[33] Net. B
ND	SA-inducible genes controlled by NPR1	7	0	[34]
NS	SA-inducible secretory genes controlled by NPR1	16	3	[34]

^a^Abbreviation for each gene network used in our study.

^b ^Genes shows the number of gene members in a given network.

^c ^*Cis *QTL is the number of the genes within the network that contain a *cis *eQTL controlling > 50% of the phenotypic variation per gene.

^d ^Reference indicates the reference source for the genes included in the network. Genes are listed in Additional file 1. ABRC refers to the use of functional network assignments as provided by the Arabidopsis Biological Resource Center [37]. The abbreviations Net. A, B, C, D and G refer to the network description provided by Reymond et al. (2004).

^e ^FVTF is *not *defined here as a network *per se*; this is a group of transcription factors that are known to affect the FV gene network. See Materials & Methods.

**Table 2:** Analysis of Variance for Network Analysis of Bay-0 Versus Sha.

Source^a^	DF^b^	SS^c^	F Value^d^	Pr > F^e^
Model	739	8796.0	81.70	< .0001
Error	220	32.0		
Total	959	8828.1		

R-Square	Coeffient of Variation (%)			
0.996369	4.009452			
				
Source	DF	Type III SS	F Value	Pr > F

NETWORK	19	4028.9	1455.4	< .0001
GENE(NETWORK)	220	4435.0	138.4	< .0001
ACCESSION	1	0.2	1.8	0.1816
REPLICATE	1	0.1	1.2	0.2688
NETWORK × ACCESSION	19	15.7	5.7	< .0001
GENE(NETWORK) × ACCESSION	220	259.6	8.1	< .0001
ACCESSION × REPLICATE	1	0.2	1.2	0.2719
NETWORK × REPLICATE	19	4.3	1.6	0.0667
GENE(NETWORK) × REPLICATE	220	49.2	1.5	0.0008
NETWORK × ACCESSION × REPLICATE	19	3.1	1.1	0.3409

^a ^Source of Variation in linear additive model.

^b ^DF = degrees of freedom.

^c ^SS = Sums of squares obtained from ANOVA.

^d ^F Value obtained from ANOVA.

^e ^Pr > F = the probability that the F value is equal to the value shown. A probability less than 0.05 indicates that the F value is significant.
